# Hookworm infection in infants: a case report and review of literature

**DOI:** 10.1186/s13052-021-00981-1

**Published:** 2021-02-09

**Authors:** G. Umbrello, R. Pinzani, A. Bandera, F. Formenti, G. Zavarise, M. Arghittu, D. Girelli, A. Maraschini, A. Muscatello, P. Marchisio, S. Bosis

**Affiliations:** 1 Università degli Studi di Milano, Milan, Italy; 2 Fondazione IRCCS Ca’ Granda Ospedale Maggiore Policlinico, Milan, Italy; 3 Department of Infectious–Tropical Diseases and Microbiology, IRCCS Sacro Cuore Don Calabria Hospital, Verona, Negrar Italy; 4 Department of Veterinary Medicine, University of Cambridge, Cambridge, UK; 5 Tropical Pediatric Unit, IRCCS Ospedale Sacro Cuore Don Calabria, Verona, Negrar Italy; 6 Laboratory of Clinical Chemistry and Microbiology, ASST Melegnano and Martesana, Milan, Italy

## Abstract

**Background:**

Hookworm infections (*Necator americanus, Ancylostoma duodenale*) are common in rural areas of tropical and subtropical countries. Human acquisition results from direct percutaneous invasion of infective larvae from contaminated soil.

Overall, almost 472 million people in developing rural countries are infected. According to simulation models, hookworm disease has a global financial impact of over US$100 billion a year. Hookworm infection in newborn or infancy is rare, and most of the cases reported in literature are from endemic countries.

Here, we describe the case of an infant with an *Ancylostoma duodenale* infection and review the literature currently available on this topic.

**Case presentation:**

An Italian 2-month-old infant presented with vomit and weight loss. Her blood exams showed anemia and eosinophilia and stool analysis resulted positive for hookworms’ eggs, identified as Ancylostoma duodenale with real time-PCR. Parasite research on parents’ stools resulted negative, and since the mother travelled to Vietnam and Thailand during pregnancy, we assumed a transplacental transmission of the infection. The patient was treated successfully with oral Mebendazole and discharged in good conditions.

**Discussion:**

Hookworm helminthiasis is a major cause of morbidity in children in the tropics and subtropics, but rare in developed countries.

Despite most of the patients is usually asymptomatic, children are highly exposed to negative sequelae such as malnutrition, retarded growth and impaired cognitive development. In infants and newborns, the mechanism of infection remains unclear. Although infrequent, vertical transmission of larvae can occur through breastfeeding and transplacentally. Hookworm infection should be taken into account in children with abdominal symptoms and unexplained persistent eosinophilia. The treatment of infants infected by hookworm has potential benefit, but further studies are needed to define the best clinical management of these cases.

## Background

Hookworms are nematode parasites responsible for different clinical disorders. They belong to the helminthic family *Ancylostomatidae*, a part of the *Strongyloidea* superfamily*.* Human acquisition occurs via ingestion of eggs and/or skin contact with infectious larvae in moist contaminated soil, therefore in public health terms they are known as soil-transmitted helminths (STH) [1]. Worldwide, *Ancylostoma duodenale* and *Necator americanus* are the two primary intestinal species, but more recently *Ancylostoma ceylanicum* has emerged as an important human parasite in some countries [2].

The prevalence of hookworm infection is affected by climatic and socioeconomic factors and represents a significant burden for public health in tropical and subtropical countries [3]. Overall, almost 472 million people in developing rural countries are infected, with the vast majority of cases occurring in South-East Asia and Sub-Saharan Africa [1, 4]. According to simulation models, hookworm disease is globally responsible for > 4 million disability-adjusted life years (DALY) lost annually, with a financial impact of over US$100 billion a year [5].

Infection prevalence typically rises with increasing age and reaches a plateau in young adults, indeed evidence from China and Southeast Asia shows that the highest prevalence is found among middle-aged subjects [6]. In the pediatric population, hookworm infection typically spreads among children when they start to crawl or walk. In newborn or infancy, on the contrary, it is rare, and most of the cases reported in literature are from tropical and subtropical countries, especially Nepal, China, India, and Africa [7–10].

Here we describe the case of a healthy Italian 2-month-old infant with an *Ancylostoma duodenale* infection. We also review literature reports of hookworm infection in children, in order to discuss the main clinical findings and the diagnostic and therapeutic approach for this condition.

## Case presentation

A two-month-old, Italian, exclusively breast-fed female was admitted to our Emergency Room for vomiting and weight loss. There was no history of fever, jaundice or abdominal distension.

One month before the admission, she was hospitalized for the same symptoms in a different Hospital. At that time, she was tested with blood and urine culture, blood exams including ammonium concentration, abdomen ultrasound and Rotavirus/Adenovirus faecal antigens. All the investigations were negative, except for the detection of eosinophilia (eosinophils count: 2900/μl). During the first hospitalization, she developed a mild diarrhoea, which was treated with probiotics, and after 4 days she was discharged in good health condition.

Upon presentation to our Clinic, the parents reported a 70 days-journey to South-East Asia (Vietnam and Thailand) during the first trimester of pregnancy, where the mother had presented several episodes of nausea and vomiting, attributed to pregnancy.

On physical examination, the infant was hemodynamically stable, alert and appropriate for age. She was well appearing and well-nourished. The abdomen was soft, and the rest of systemic examination was within normal limits. Physical findings were as follows: body weight 4.75 kg; height 60 cm; heart rate 120 beats/min; body temperature 36 °C; oxygen saturation in room air 96%; blood pressure 70/52 mmHg. On admission to our Hospital, the laboratory tests showed a white blood cell count of 19,060/μl with 27.1% of eosinophils (5170/μl), platelets count 756,000/μl, haemoglobin 9.1 g/dl, peripheral smear showed microcytic hypochromic red blood cells (RBCs). Given the hemoglobin levels, an oral supplementation with iron and folic acid was started. Electrolytes, renal function and coagulation tests were within the normal range. Serum proteins, albumin, bilirubin and liver enzymes were normal; C-reactive protein (CRP) was negative. The abdomen ultrasound was normal; urinary culture was negative. Grossly, stools looked normal, with no blood appearance or melena; viral antigen and bacterial culture were negative.

**Fig. 1:**
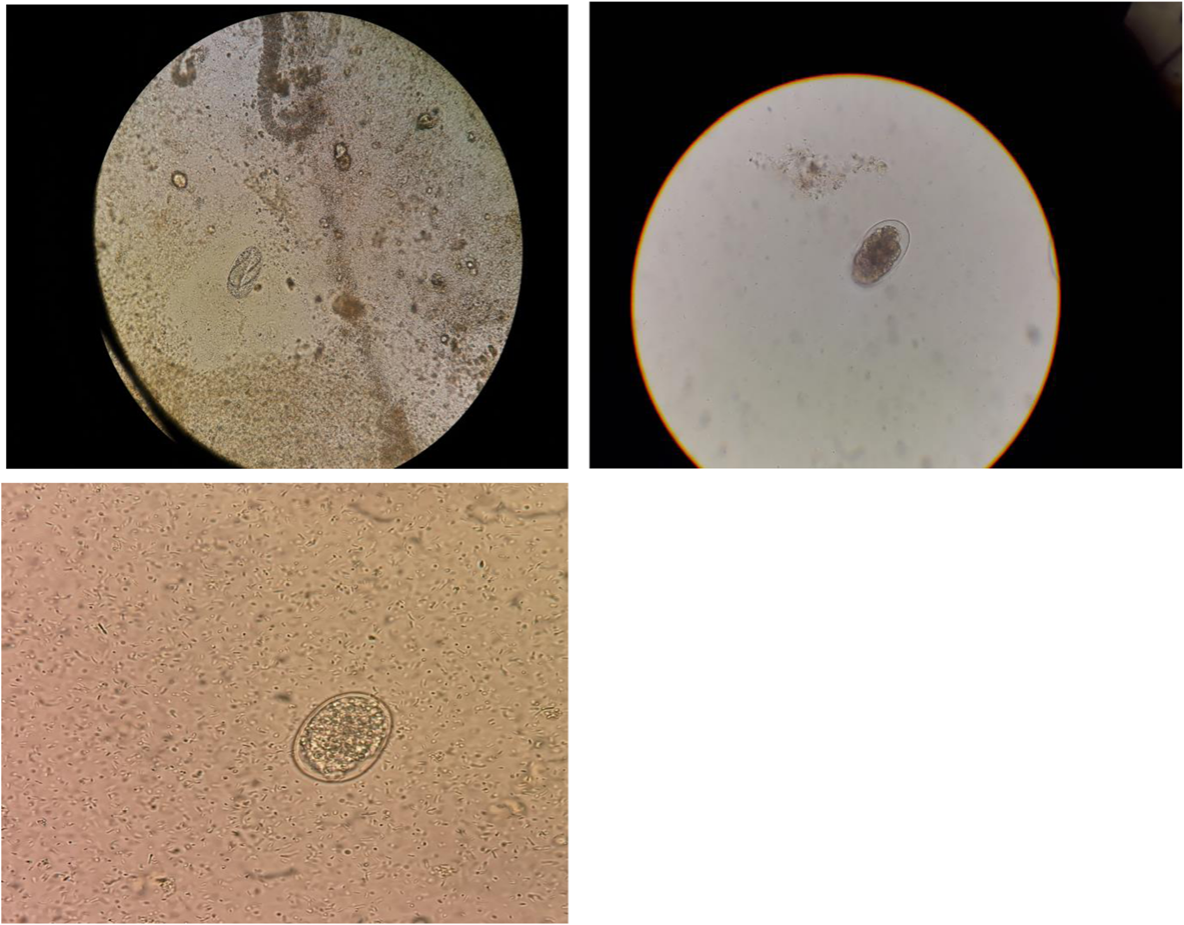
Fresh microscopical analysis of the stools showing eggs of *Ancylostoma duodenale*

The microscopic examination of stools detected the presence of hookworm’s eggs which were confirmed in three different samples from different days (Fig. 1). The collected specimens were subsequently sent to the specialist parasitology laboratory of “Ospedale Sacro Cuore Don Calabria” of Negrar, where were tested with molecular biology techniques (Real Time polymerase chain reaction, RT-PCR) which confirmed the species as *Ancylostoma duodenale*. Parasitic research on parents’ stools resulted negative, suggesting a possible vertical transmission of *Ancylostoma* infection during pregnancy. The infant was treated with oral Mebendazole (100 mg/die in two doses) for 3 days, which was well tolerated, without any complications. She was discharged in good general conditions.

At a follow-up visit after 1 month, her clinical presentation was normal; blood exams were normal with resolution of anemia and normalization of eosinophils and platelets count, and stool microscopic examination was negative.

## Discussion

Hookworm human infection is a public health problem, especially among rural areas of developing countries. The burden of hookworm infection is primarily related to the impact on health and quality of life. In infancy this is the consequence of a chronic infection, which may adversely affect growth, nutritional status and cognitive capacity [11].

After the direct percutaneous invasion of infective larvae from contaminated soil, at the end of their life cycle, adult worms parasite the upper small intestine and use cutting organs to suck arteriolar and capillary blood of the intestinal mucosa. In moderate and severe disease, this can lead to iron-deficiency anemia, particularly in high-risk subjects, such as young women of child-bearing age and children [6, 12].

While most of the cases are asymptomatic, occasionally the infection can cause overt gastrointestinal bleeding and maelena, as reported also in infants from endemic areas [7, 13]. Long term consequences are both iron deficiency anemia and protein malnutrition, with subsequent growth stunting and decreased cognitive capacity, especially working memory [14]. Among the other classical manifestations there are gastrointestinal disorders, as abdominal pain, tenderness, diarrhea and vomiting. Less commonly hookworms may cause ground itch and eosinophilic pneumonia [1].

Hookworm helminthiasis remains a major cause of morbidity in infants and children in the tropics and subtropics. In contrast, it is rarely described in developed countries, and little is known about its incidence in the Italian population [15].

These infections are considered uncommon in local children, but only few studies had reported epidemiological data on the diffusion of intestinal parasitic disease Italian country, always considering together children and adults [16–20].

According to a six-years retrospective analysis performed in Ancona on 5323 adults and children [16], helminths were found in 0.9% of the subjects, with a higher prevalence in children, both in Italian and non-Italian patients. *Hymenolepis nana*, *Strongyloides stercoralis* and *Trichuris trichiura* were the most common species identified in non-Italian children, suggesting that certain parasites are restricted to endemic tropical areas. Another study [17] conducted on 5351 patients over a two-years period in Rome found a similar prevalence of helminth infections (0,5%), higher in non-Italian subjects, considering both children and adults. Infection rate was low in children under 5 years of age, reaching a pick between 5 and 14 years and then declining progressively.

Overall, these findings confirm that intestinal parasites are not limited to endemic areas but can be found also in Italian country, although uncommon in natives. In support of this evidence, a recent Italian study [21] involving 584 internationally adopted children showed that intestinal parasites represent a widespread infection in these subjects, especially in school-age children. Data analysis revealed a positive association between parasitic infection and age – with a higher prevalence in children between 2 and 5 years old, that increased over 5 years of age – country of origin and eosinophilia.

According to literature helminthic infections are uncommon in local children, anyway, they should be suspected at any age, especially when clinical findings are supported by epidemiological data. Indeed, their presence in children in Italy can be explained by the increased number of travels abroad, imports of food, immigration, and adoptions.

In newborns and infants, the exact mechanism of infection still remains unclear. For those subjects who become symptomatic before the age of 5–8 weeks, a vertical transmission should be considered. Indeed, clinical reports from endemic countries assess the possibility of a vertical transmission of dormant larvae through breastfeeding or rarely transplacentally [8]. Conversely, in older children, the infection is often acquired from contaminated soil, or occasionally through larva-infested waters [10].

Although we cannot be certain of the route of contamination in our patient, it is unlikely that the infant acquired it from the environment. In fact, she lived in Italy since her born, she was from a high socioeconomic background and we were not able to detect the presence of parasites in parents’ stools. The family had no accompanying animals and given her age, the acquisition through contaminated soil seemed unlikely as well.

Considering the history of a travel in an endemic country during pregnancy and the concomitant occurrence of gastrointestinal symptoms in the mother, we assumed a transplacental transmission of *Ancylostoma duodenale*.

In our case, the patient showed a persistent increase in eosinophil counts on peripheral blood. This occurs with worms that have continuous tissue invasion and contact with immune effector cells, such as hookworms [14]. Therefore, an unexplained persistent eosinophilia may be a major clue for the presence of a parasitic infection and could justify specific investigations on stools.

Ova/larvae detection in stools is diagnostic, but the correct nematode identification may be challenging. In fact, microscopical diagnosis is influenced by the type of organism, its life cycle and the amount of egg or larval shedding [22]. Furthermore, the eggs of *A. duodenale* and *N. americanus* are indistinguishable at the microscopic observation [3]. Molecular methods can overcome these potential obstacles. In particular, RT-PCR provides an increased detection rate and a significantly higher specificity, enabling the distinction between the different species [22], which is helpful also to choose the appropriate therapy, considering the potential variation in the response to chemotherapeutic intervention [23].

Literature provides little information about the use of antielminthic drugs under 24 months of age. Globally, the use of benzimidazoles with different protocols (albendazole 400 mg single dose, or mebendazole 500 mg single dose, or mebendazole 100 mg twice daily for 3 days) is recommended for individual treatment [1], although more doses of albendazole may be needed to control the infection [24]. Sides effects of these drugs are rare and mostly represented by abdominal pain, nausea and diarrhea; however, the safety in children under 12 months has not been established yet [1]. A recent study on the use of mebendazole in 45 breastfeeding mothers found no adverse effects in infants [25], but so far only a few studies investigated the effects of its use directly on infants [26]. Current knowledge shows that the incidence of side effects linked to benzimidazole drugs in young children is likely to be the same as in older children and adults [27]. Therefore, in light of the potential benefit on physical and cognitive development, the treatment of young infants could be justified. Anyway, wider studies are needed to confirm these data.

In conclusion, although hookworm infections are infrequent in developed countries, they should be taken into account in children with abdominal symptoms and unexplained persistent eosinophilia. A vertical transmission has been postulated for newborns and infants, and a history of travel in an endemic country during pregnancy should always be investigated. Morphological identification of hookworms is difficult, therefore molecular methods could be useful tools for the correct diagnosis. Currently, no guidelines are available for the treatment and the follow-up of young infants. Despite the potential benefit of a treatment, further studies are needed to define the best clinical management of these cases.

## Data Availability

Not applicable.
